# Synthesis of Magnetic Fe_3_O_4_ Nano Hollow Spheres for Industrial TNT Wastewater Treatment

**DOI:** 10.3390/nano12050881

**Published:** 2022-03-07

**Authors:** Shafi Ur Rehman, Sana Javaid, Muhammad Shahid, Mutawara Mahmood Baig, Badar Rashid, Caroline R. Szczepanski, Sabrina J. Curley

**Affiliations:** 1 School of Chemical and Materials Engineering (SCME), National University of Sciences and Technology (NUST), Islamabad 44000, Pakistan; shafi.phdscme@student.nust.edu.pk (S.U.R.); mutawara.phdscme@student.nust.edu.pk (M.M.B.); 2 School of Natural Sciences (SNS), National University of Science and Technology (NUST), Islamabad 44000, Pakistan; sana.javaid@sns.nust.edu.pk; 3 Department of Chemistry, University of Wah, Quid Avenue, Wah Cantt, Rawalpindi 47040, Pakistan; 4 Dean of Research and Development (R & D), National University of Technology NUTECH, Islamabad 44000, Pakistan; seebadar@yahoo.com; 5 Department of Chemical Engineering & Materials Science, Michigan State University, East Lansing, MI 48824, USA; szcz@msu.edu (C.R.S.); curleysa@msu.edu (S.J.C.)

**Keywords:** magnetite (Fe_3_O_4_), TNT, nano hollow spheres NHS, wastewater treatment

## Abstract

The aim of the present work was to synthesize magnetite (Fe_3_O_4_) nano hollow spheres (NHS) via simple, one-pot, template-free, hydrothermal method. The structural, morphological, and surface analysis of Fe_3_O_4_ NHS were studied by scanning electron microscopy (SEM), x-ray diffraction technique (XRD), Fourier transform infrared spectroscopy FTIR and burner-Emmett-teller (BET). The as obtained magnetic (Fe_3_O_4_) NHS were used as an adsorbent for treating industrial trinitrotoluene (TNT) wastewater to reduce its Chemical Oxygen Demand (COD) values. Adsorption capacity (Qe) of the NHS obtained is 70 mg/g, confirming the attractive forces present between adsorbent (Fe_3_O_4_ NHS) and adsorbate (TNT wastewater). COD value of TNT wastewater was reduced to >92% in 2 h at room temperature. The adsorption capacity of Fe_3_O_4_ NHS was observed as a function of time, initial concentration, pH, and temperature. The applied Fe_3_O_4_ NHS was recovered for reuse by simply manipulating its magnetic properties with slight shift in pH of the solution. A modest decrease in Qe (5.0–15.1%) was observed after each cycle. The novel Fe_3_O_4_ NHS could be an excellent candidate for treating wastewater generated by the intermediate processes during cyclonite, cyclotetramethylene-tetranitramine (HMX), nitroglycerin (NG) production and other various environmental pollutants/species.

## 1. Introduction

2,4,6-Trinitrotoluene (TNT) is a versatile aromatic compound used in drugs, herbicides, insecticides, dyes, polyurethane foams, and fungicides [1,2,3]. It is one of the most conventional explosives in use since the late 19th century, known for its insensitivity to shock and friction. Its influence is so pervasive that the standard unit for measuring the energy released after a detonation is a “ton of TNT,” equaling 4.184 gigajoules. This metric aids in measuring the strength of bombs, detonation velocities and penetration power of other explosives [4,5,6].

When synthesizing TNT, the washing step during manufacturing produces waste products that end up in the surrounding environment, both in soil and particularly in water streams. These include dissolved species such as sulfates, mono nitro toluene (MNT), di nitro toluene (DNT), dinitro toluene sulfonate (DNTS) and several other derivatives of nitrobenzene (NB) [7]. These nitrogenous compounds exist in TNT wastewater in different concentrations, resulting in discoloration of the wastewater (e.g., red water, orange water, yellow water, etc.). The exact hue of the discoloration depends on the intensities of the nitro and sulphonates groups present [8]. TNT wastewater exhibits the highest values of chemical oxygen demand (*COD*), ranging from 600 to 6000 mg/L depending on the amount of these different aromatic compounds dissolved during the washing process of TNT production. It was observed that the intensity of TNT and other associated aromatic compounds in wastewater are a predominant factor impacting the local ecosystem [9]. Following the guidance issued by the Environmental Protection Agency (EPA), TNT wastewater cannot be released in nearby streams, lakes or outside industrial premises without proper treatment [10]. The presence of MNT, DNT, sulfonates and other nitrobenzene derivatives causes the soil and water to become increasingly more toxic and hazardous [11].

Generally, proper and effective treatment for TNT wastewater entails either the degradation or adsorption of these compounds [12,13,14,15,16]. Although MNT and DNT can be treated through wet air oxidation and reduction processes, the application of these treatments on a large scale has a high associated cost. As a result, other avenues which are simpler, and more cost-effective must be explored. Adsorption processes are ultimately more convenient to reduce *COD* as they are already widely employed in the form of silica, activated carbon and different resins [17,18,19]. TNT wastewater and many more industrial effluents have also been treated with catalysts such as microporous polystyrene resin RS 5OB, granular activated carbon (GAC) and nanoscale zerovalent iron particles (NSZi) through a combination of advanced oxidation and Fenton process, etc. [20,21,22,23,24]. Polystyrene resin RS 5OB can reduce *COD* values of TNT wastewater, however, this comes at the cost of high resin consumption and requires a high dose rate, approximately 120 g/L or greater. A significant amount of time is also required for adsorption, typically 8–10 h, which makes such resins unsuitable for continuous processes, such as treating TNT wastewater. Furthermore, the resin is relatively expensive and requires tedious chemical treatments to recycle it for reuse. Granular-activated carbon was also applied for adsorption of TNT and its derivatives dissolved in wastewater with certain limitations [22]. Mainly, with the strategy, it was necessary to maintain the temperature above 60 °C. Additionally, the flow rate and pH must also be maintained within specific limits (1.0 g/L and a pH of 2.0) to achieve the optimum adsorption rate. Using activated carbon, only a low concentration of TNT wastewater (e.g., less than 60 ppm) can be treated. However, in industrial TNT washing, the concentration of TNT and its derivatives likely exceeds 60 ppm, thus limiting the applicability of activated carbon as an approach.

Nanoscale zerovalent iron particles are another promising catalyst employed to address TNT wastewater [25,26]. This method is more effective at adsorbing di-substituted toluene, e.g., Di-nitro Toluene Sulphonates (DNTS) and has the capability to convert DNTS into corresponding di-amino toluene sulfonates. This process can be applied for specific adsorption of DNTS within a narrow range of pH values, e.g., neutral or near to neutral values (pH 5–7). In a recent study, Brazilian TNT industrial wastewater was treated using nanoscale zerovalent iron particles, where the zero-valent iron and Fenton process were coupled together [23]. This coupled method was found to be more effective towards the removal of the absorbed species and *COD* decrease, (e.g., *COD* < 90%). The values of *COD* and the actual toxicity were significantly reduced. Unfortunately, this coupled technique has a high associated cost, and thus scale-up remains a challenge [27,28].

To combat these limitations, magnetic nanoparticles (MNPs), which have captured the attention of many researchers in wastewater treatment/purification could be a better option. These MNPs possess high surface area, a greater surface-to-volume ratio, specificity, pore size effect and paramagnetic behavior (Although there are several types of MNPs, e.g., metal oxides (Fe_2_O_3_, Fe_3_O_4_), metals (Ni, Co, Fe), spinal-type antiferromagnets (MgFe_2_O_4_, MnFe_2_O_4_, CoFe_2_O_4_), and alloys such as CoPt, FePt and FePd, only iron oxides are benign and resist oxidation in biological systems. Magnetic nanomaterials such as zero-valent iron, hematite (α-Fe_2_O_3_) and magnetite (Fe_3_O_4_) have a multitude of applications in molecular biology, medicine, degradation of dyes, drug delivery and remediation of industrial polluted water [29,30,31,32].

Due to the adsorption, degradation, and magnetic properties of Fe_3_O_4_, it has become a useful tool for wastewater/effluent treatment [33,34]. Many researchers have utilized Fe_3_O_4_ to adsorb dissolved species in industrial wastewater [23]. Nagi et al. applied nano-spherical quantum dots of Fe_3_O_4_ to remove heavy metals such as Cr, Co, and pesticides [35]. Additionally, Elhassan et al. exploited the adsorption behavior of magnetite along with other nano metal oxides with components such as Cu^2+^, Pb^2+,^ Cr^+4^, Cd^2+^ and Ni^2+^, to remove atrazine and bisphenol-A from wastewater [36]. Ali Nematollah Zadeh et al. also reported the application of modified (Fe_3_O_4_) nanoparticles for the adsorption of nitro benzene (NB), with a reported adsorption capacity of 66.72 mg g^−1^ [37]. Modified Fe_3_O_4_ nanoparticles were used by Meiling et al. for the detection of heavy metal ions in water and successful detection of Cu^2+^, Cd^2^, Zn^2+^ and Hg^2+^ were carried out in an aqueous solution and applied further for purification of water [38]. The structural and magnetic properties of ZnxFe_3_−xO_4_ nano hollow spheres were also investigated by Priyanka Saha et al., in which Zn was doped in magnetic nano hollow spheres (NHS) and tested successfully for biomedical applications [39]. Mahmood Iram et al. synthesized Fe_3_O_4_ (NHS) through the hydrothermal method, which could then be employed for successful adsorption of Natural Red Dye [26]. Xiang Wang et al. reported the magnetic nanocomposites of Triethylenetetramine-modified Fe_3_O_4_/SiO_2_/CS-TETA for adsorption of Cr (VI). These researchers observed an adsorption capacity for Cr (VI) ions as high as 254.6 mg g^−1^ along with remarkable adsorption equilibrium times as less as 15 min [40]. However, magnetite has never been tested on TNT wastewater adsorption, to the best of our knowledge so far.

The novelty of the present work is the synthesis of the α-Fe_2_O_3_ (hematite, rhombohedral crystal structure, R_3_ c) and Fe_3_O_4_ (magnetite, face-centered cubic crystal (FCC) structure, Fm3 m) through a hydrothermal, template-free method, using the same precursors while exploring modifications in temperature and calcination time. The economic factor in this work is the reuse of the applied catalyst (Fe_3_O_4_ NHS), after recovering it by simply altering the pH of the solution and using its magnetic properties.

Fe_3_O_4_ (NHS) was employed as an adsorbent to investigate the adsorption efficacy and reduction in COD values of TNT wastewater. The mechanism for the removal of nitro bodies, sulfates, and other derivatives of nitro benzene present in the sample proceeds via an ion-exchange mechanism among positively-charged TNT water molecules and OH- at the Fe_3_O_4_ catalyst surface. The fine control over phase purity and crystallinity of the iron oxides during synthesis are the major challenges with the method employed. However, our method for synthesizing iron oxides in an aqueous solution does have the advantage of reduced cost, being environmentally friendly and associated minimum chemical waste and energy consumption.

## 2. Materials and Methods

### 2.1. Materials

All chemical products were purchased from Sigma Aldrich, Germany supplied by Pro—Marketing Company Islamabad, Pakistan. All the precursors used in this work were of analytical grade with high purity (≥98%) including cetyl trimethyl ammonium bromide [CTAB], potassium ferricyanide K_3_ [Fe (CN)_6_], ammonium per sulfate (NH_4_)_2_S_2_O_8_, sodium di-hydrogen phosphate [NaH_2_PO_4_] and ethanol.

#### Adsorbent

**Figure 1:**
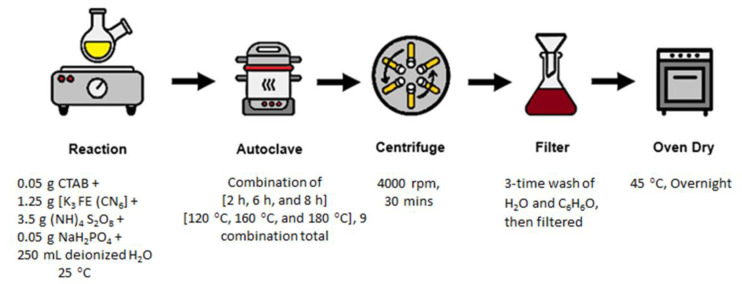
Schematic of the synthetic strategy employed to synthesize α-Fe_2_O_3_ and Fe_3_O_4_ (NHS), with synthetic parameters for each step listed.

For the synthesis of Fe_3_O_4_ (NHS), 0.05 g of CTAB, 1.25 g of [K_3_ Fe (CN)_6_], 3.5 g of (NH_4_)_2_S_2_O_8_, and 0.05 g of 0.02 M NaH_2_PO_4_ solution were dissolved in 250 mL de-ionized water. The solution was magnetically stirred at room temperature in the presence of nitrogen gas until the solution turned visibly yellow in color. The solution was then poured into a 250 mL Teflon cup, sealed properly in an autoclave, and kept for calcination in an oven for 2 h, 6 h, and 8 h at a temperature of 120 °C, 160 °C and 180 °C, respectively. The impact of these process parameters on the shape and morphologies of the catalyst was determined. After the completion of hydrothermal treatment, the solution was centrifuged at 4000 rpm for 30 min and washed three times with de-ionized water and ethanol systematically. The dark brown solution was filtered and then dried in a vacuum oven overnight at 45 °C (Figure 1).

### 2.2. Characterization Techniques

#### 2.2.1. Scanning Electron Microscopy (SEM)

Surface morphologies of the as-obtained products after varying time and temperature conditions were studied using Scanning Electron Microscopy (Modal JSM 6490LA, JEOL, Tokyo, Japan) at 20 kV.

#### 2.2.2. X-Ray Diffraction (XRD)

Structural analysis of the samples was performed with an X-ray diffractometer (Model: X’ TRA48 Thermo ARL, Tokyo, Japan using Cu Kα radiation (k ¼ 0.15406 nm), operating at 40 mA and 45 kV. The radial scans were performed in reflection scanning mode with 2θ values ranging from 5 to 80 and at a scanning rate of 1 min^−1^. The patterns were evaluated, carefully examined, and reconfirmed with the records from the International Centre for Diffraction Data (ICDD) to verify the identity of the products.

#### 2.2.3. Brunner–Emmet–Teller (BET)

BET adsorption was performed using a Surface Area and Porosity Analyzer (Model: Micromeritics Gemini VII, Norcross, GA, USA) for analyzing porosity and surface area of the synthesized Fe_3_O_4_ NHS.

#### 2.2.4. Fourier Transform Infrared Spectroscopy (FTIR)

The functional groups in the synthesized samples were investigated through Fourier Transform Infrared Spectroscopy (FTIR, Model Nicolet 6700, Thermo Scientific, Waltham, MA, USA). Samples were shaped into pellets interspersed with KBr powder, and the respective spectra were obtained using attenuated total reflectance mode in the range of 4000 to 400 cm^−1^ with a resolution of 6 cm^−1^. An average of 32 scans are reported for each sample.

#### 2.2.5. Ultraviolet/Visible Spectrophotometer (UV-Vis)

UV/visible absorbance of TNT was observed via UV-Vis spectrophotometry (Model: Jenway 630501 6300 Visible Spectrophotometer 220 V, Livingston, UK). Solutions of TNT in ppm (parts per million) for different solvents (benzene, toluene, ethanol, and methanol coded as B, T, E and M, respectively) were prepared for analysis. The performance of (Fe_3_O_4_) NHS was investigated by varying concentration, weight of the adsorbent applied, contact time and temperature.

#### 2.2.6. Chemical Oxygen Demand (*COD*) Determination

The *COD* of the TNT wastewater samples was determined by the standard method (Merck Method) [41]. For this, 50 mL of the sample was placed in a 500 mL conical flask with 50 mL distilled water, 25 mL Potassium Dichromate solution, 1.0 g silver sulfate and 2.0 g of mercury (II) sulfate. Approximately 75 mL of concentrated sulfuric acid was added dropwise under continuous stirring. The mixture was boiled over the sand bath for 2 h under reflux and then cooled for 30 min. The obtained mixture was then treated with 0.25 M ammonium iron (II) sulfate solution until the color changed from bluish-green to reddish-brown. Under the same conditions the blank sample was also determined using 50 mL distilled water instead of the TNT red water. *COD* values were calculated using the following formula.

(1) $$COD=\frac{\left(A-B\right)*C*f*8000}{D}$$

Here, A is the mL ammonium iron (II) sulfate solution titrated with blank (solvent), B is the mL ammonium iron (II) sulfate solution titrated with the sample (TNT wastewater), C is the molarity of ammonium iron (II) sulfate solution, f is the titer molarities (1 M) (from MERCK table), and *D* is the mL effluent sample (TNT wastewater) used.

The adsorption capacity (*Qe*) and efficiency (η) of Fe_3_O_4_ NHS were determined by the following formulae.

(2) $$Qe=\frac{\left(COD\right)i-\left(COD\right)e\;V}{W}$$

(3) $$\eta =\frac{\left(COD\right)i-\left(COD\right)e}{\left(COD\right)i}$$

Here, (*Qe*) and (η) are the adsorption capacity and efficiency of the Fe_3_O_4_ NHS respectively, (*COD*)*i* is the initial *COD* of TNT wastewater, (*COD*)*e* is the value of *COD* at equilibrium, *V* is the volume of TNT wastewater used and W is the weight of Fe_3_O_4_ NHS applied.

## 3. Results and Discussion

### 3.1. Scanning Electron Microscopy (SEM)

**Figure 2:**
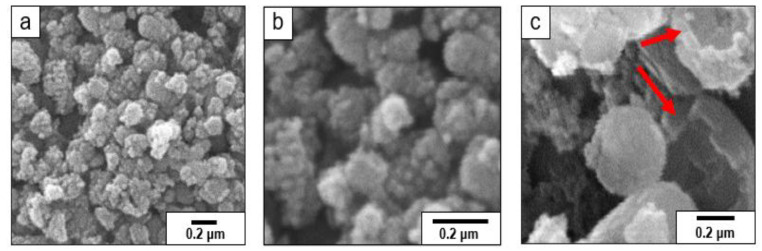
SEM images of α-Fe_2_O_3_ particles autoclaved for 2 h at 120 °C (**a**), 6 h at 160 °C (**b**), and Fe_3_O_4_ (NHS) autoclaved for 8 h at 180 °C (**c**) Red arrows point to areas where the hollow cavity of the particles can be observed in Fe_3_O_4_ (NHS).

The shape and morphology of the synthesized Fe_3_O_4_ NHS were investigated via SEM (Figure 2). A compact morphology with an average size varying from 11 to 112 nm formed after 2 h of calcination at 120 °C in an autoclave (Figure 2a). On the other hand, for our next case as the reaction progressed for 6 h at 160 °C, pores formed on the surface of the nanoparticles, increasing the surface area-to-volume ratio, shown in (Figure 2b). Calcination treatment of 8 h at 180 °C resulted in the formation of an intense porous outer surface as shown in (Figure 2c). The porous architecture of the Fe_3_O_4_ NHS enhances surface adsorption properties due to the large available surface area, thus making these NHS effective dsorbent for our targeted adsorption [30,42].

With this template-free synthesis, we concluded that the pore size, shape, volume, and surface morphologies of Fe_3_O_4_ NHS depend on the calcination time and temperature. In this work, our results show the ideal temperature and calcination time in an autoclave were (180 °C and 8 h). The concentration of Na_2_H_2_PO_4_ employed here plays an important role in the synthesis of the cavities inside the Fe_3_O_4_ NHS. This choice was motivated by prior studies indicating that with higher concentrations of Na_2_H_2_PO_4_, the acidity of the solution increases, leading to an uncontrolled rate of ionization that may destroy the magnetite structure [32]. The ionization of K_3_Fe (CN)_6_ leads to the formation of hollow spheres of Fe_3_O_4_ upon addition of Na_2_H_2_PO_4_, which brings high surface energies and associated high stability to the product [21]. Ostwald ripening is the proposed mechanism for the synthesis of these Fe_3_O_4_ NHS through the one-pot, template-free, hydrothermal method in an aqueous solution [43]. Here, nucleation and growth dominate, with large particles growing larger due to the instability of the high surface-to-volume ratio associated with small particles. Various factors affecting the Ostwald ripening process include particle size, solubility, surface energy and dissolution [44]. In this work, we employ CTAB as a dispersant to maximize the yield of Fe_3_O_4_ NHS.

### 3.2. X-Ray Diffraction Spectroscopy (XRD)

**Figure 3:**
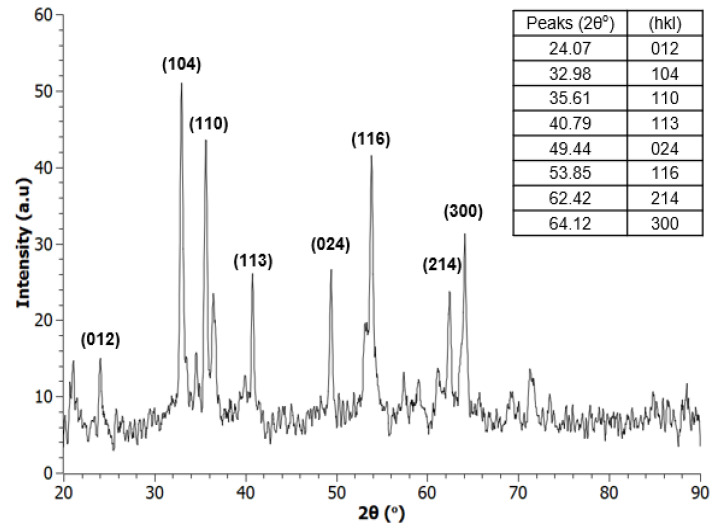
XRD spectra of α-Fe_2_O_3_ (hematite), autoclaved at 160 °C for 6 h.

**Figure 4:**
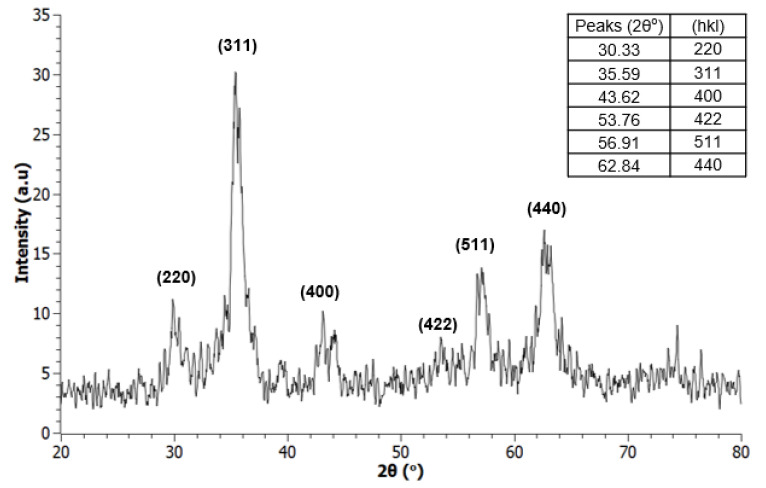
XRD spectra of Fe_3_O_4_ NHS (magnetite), autoclaved at 180 °C for 8 h.

XRD analyses were carried out to confirm the phase purity of the as-obtained samples and identify the iron oxide from the various phases possible. For this purpose, reaction conditions and calcination times were optimized as discussed earlier, providing both desired α-Fe_2_O_3_ (hematite, rhombohedral crystal structure, R3 c) and Fe_3_O_4_ (magnetite, face-centered cubic (FCC) crystal structure, Fm3 m) products as shown in Figure 3 and Figure 4. The XRD data obtained were checked against standardized records from the International Centre for Diffraction Data (ICDD) to verify the identity of the products.

### 3.3. Brunner–Emmett–Teller (BET) Adsorption Method

**Figure 5:**
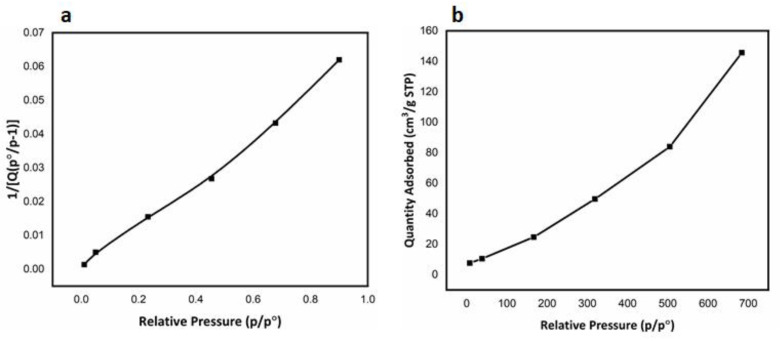
(**a**) BET surface area plot and (**b**) Langmuir surface area plot of Fe_3_O_4_ NHS showing the intent of N_2_ adsorption over the surface of Fe_3_O_4_ NHS upon increasing relative pressure.

The adsorption behavior of Fe_3_O_4_ NHS over nitrogen gas was measured, as shown in Figure 5 to determine the efficacy of this adsorbent for TNT wastewater treatment. The BET graph shows the relationship between the adsorption of N_2_ gas (1/[X (P^0^/P)−1]) and the relative pressure (P/P^0^) applied, showing a positive linear behavior which indicates an improved rate of adsorption with increasing relative pressure. The results we obtained from the BET tests are encouraging and thus provide us with the required data for a Fe_3_O_4_ NHS to be applied on TNT wastewater as an adsorbent tool [45,46].

The surface area and pore size of the Fe_3_O_4_ NHS were greater than that of α-Fe_2_O_3_. Fe_3_O_4_ NHS produced under the conditions (8 h at 180 °C) had a BET surface area of 66.057 m^2^/g, with a calculated Langmuir surface area of 650.288 m^2^/g, and a cumulative surface area of 144.096 m^2^/g. The average pore volume calculated is 0.225 cm^3^/g and the pore size is 136.429 A°. The tabulate data for BET isotherm and BET surface area are shown in Table 1 and Table 2, respectively.

The surface area of the adsorbent obtained here is due to the face-centered cubic (FCC) interstitial spaces between the adjacent Fe_3_O_4_ NHS. However, fine control over the size and pore volume of Fe_3_O_4_ NHS thus remains a challenge using this synthetic route. The rate of ionization of [Fe (CN)6−3] upon addition of [H_2_PO_4_−] plays an important role in managing the overall acidity of the ongoing reaction. Ultimately, the hollow porous architectures formed here consist of both micro and nano-sized spheres. Despite these variations in particle size, the mesoporous architecture leads to enhance photocatalytic activity and effective adsorption of organic species from TNT wastewater and straightforward recovery via a simple magnetic separation method.

### 3.4. Fourier Transform Infrared Spectroscopy (FTIR)

**Figure 6:**
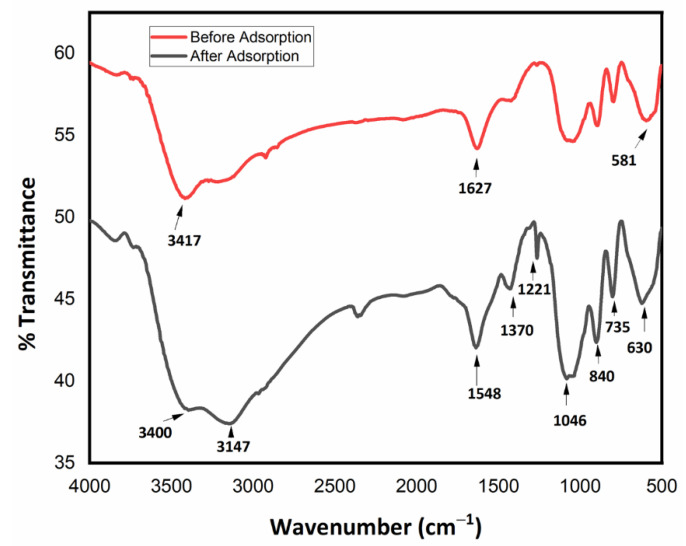
FTIR spectra of Fe_3_O_4_ NHS before and after its application on TNT wastewater for required adsorption.

**Figure 7:**
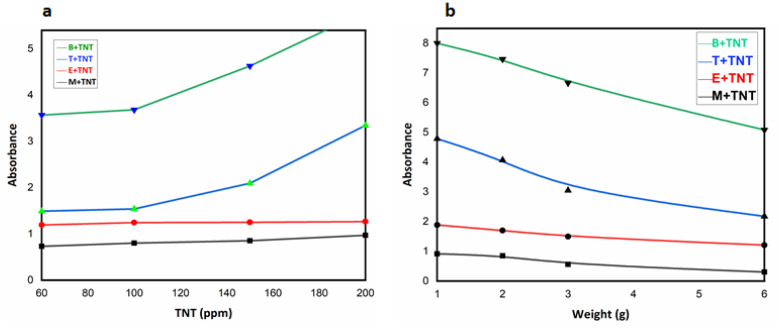
(**a**) Effect of increasing TNT concentration on absorbance (**b**) Effect of increasing catalyst (Fe_3_O_4_ NHS) dosage on absorbance at room temperature (25 °C).

FTIR characterization of the Fe_3_O_4_ NHS before and after its interaction with TNT wastewater is reported in Figure 6. The stretching and bending vibrations of Fe_3_O_4_ NHS closely resemble the standard spectrum reported in prior works [20,34]. The adsorption peak present at 581 cm^−1^ refers to the characteristic peak of (Fe–O). Hydroxyl group (O–H) bending and stretching vibrations are observed at 1627 cm^−1^ and 3417 cm^−1^, respectively, in the neat Fe_3_O_4_ NHS sample spectra before adsorption. FTIR spectra were also collected after applying the Fe_3_O_4_ NHS to the TNT wastewater sample (Figure 7, black spectra). The result clearly shows characteristic peaks of some nitro and sulfate groups which were adsorbed to the surface of the Fe_3_O_4_ NHS Fe_3_O_4_ NHS. Specifically, peaks at 1548 cm^−1^ and 1370 cm^−1^ are attributed to the asymmetric and symmetric vibrations of the nitro groups adsorbed by the Fe_3_O_4_ NHS [47]. Similarly, the asymmetric and symmetric stretching of sulfonates groups are observed at 1221 cm^−1^ and 1046 cm^−1^, respectively [48]. Additionally, stretching vibration of (C–N) bond at 840 cm^−1^ and scissoring vibration of nitro groups at 735 cm^−1^ are observed. Furthermore, the bending vibration of (C–N = O) group is also observed at 630 cm^−1^ [21]. With the above observations, it is confirmed that the Fe_3_O_4_ NHS applied on TNT wastewater has adsorbed the derivatives of nitrobenzene including 2,4-DNT-3-SO-3 and 2,4-DNT-5-SO-3 from the TNT wastewater and thus decreased the *COD* values, as per our expectations.

### 3.5. Ultraviolet/Visible Spectrophotometer (UV-Vis)

UV/Visible absorbance for the TNT solutions shows the trend of increase in absorbance with increasing TNT concentration in different solvents (benzene, toluene, ethanol, and methanol coded as B, T, E and M, respectively) as shown in Figure 7a. This illustrates how the quantity of dissolved TNT increases the UV/visible absorbance of the sample, following the Beer–Lambert law [49]. The performance of the synthesized Fe_3_O_4_ NHS in terms of decreasing UV/visible absorbance is shown in Figure 7b. Here, the gram amount of the adsorbent added to different TNT solutions was varied. Continuous, magnetic stirring (350 rpm) was employed to expose the high surface area of the Fe_3_O_4_ NHS to TNT molecules present in solution and thus facilitate their adsorption at room temperature (25 °C).

**Figure 8:**
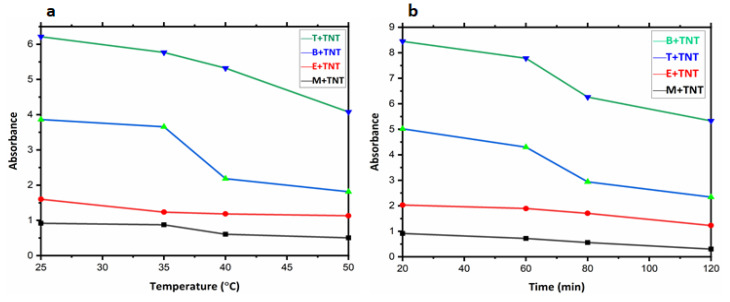
(**a**) Decrease in absorbance as a result of increase in temperature of TNT solutions (1.0 g Fe_3_O_4_ NHS, 60 ppm TNT solution), (**b**) Effect of contact time between adsorbate and adsorbent result in decrease in absorbance for TNT solutions (1.0 g Fe_3_O_4_ NHS).

Within the limits associated with the volatilities and temperature limits of the organic solvents used here, the TNT solutions were exposed to heat to determine the influence of temperature on UV/visible absorbance (Figure 8a). It was observed that with increasing temperature, the kinetic energy also increases, maximizing the accommodation of nitro bodies over the surface area of the adsorbent which leads to a decrease in UV/visible absorbance (Figure 8a).

Contact time between the adsorbate and adsorbent is a vital part in determining the overall efficiency of the adsorbent applied for adsorption in any industrial process. This important process parameter was also measured for Fe_3_O_4_ NHS synthesized here, by increasing contact time up to 2 h. while maintaining a constant amount of adsorbent (1.0 g). The influence of contact time on UV/Visible data is shown in (Figure 8b). Increasing the contact time between the Fe_3_O_4_ NHS and TNT solution ultimately provided the adsorbent enough time to impregnate its surface and active sites with adsorbate molecules and thus results in optimized adsorption.

### 3.6. Chemical Oxygen Demand (COD)

**Figure 9:**
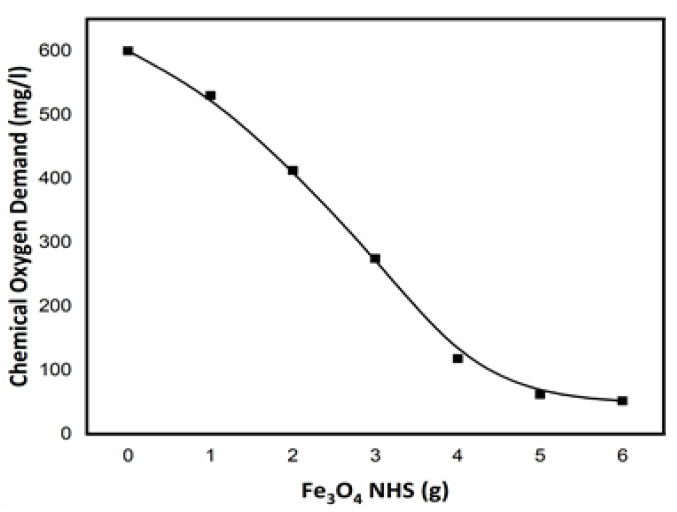
Effect of Fe_3_O_4_ NHS concentration over COD reduction using MERK method at room temperature (25 °C).

**Figure 10:**
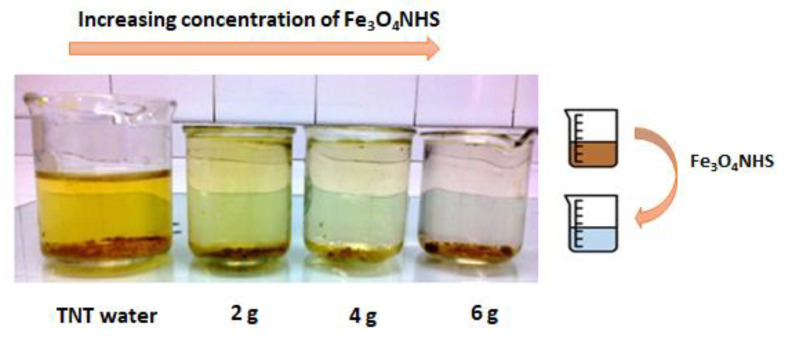
More toxins in the TNT wastewater are bound to the surface of Fe_3_O_4_ NHS added to the mixture. This yields clearer water with increasing Fe_3_O_4_ NHS present.

A *COD* test was carried out using the MERCK method for TNT wastewater samples provided by Pakistan Ordnance Factories (POFs) Wah Cantt, Pakistan [41]. To begin, 1.0 g of Fe_3_O_4_ NHS was added to 50 mL of the TNT wastewater samples for 2 h. The initial *COD* of this sample was calculated as 600 mg/L using (Equation (1)). A linear decline in *COD* values was observed with increasing amount of Fe_3_O_4_ NHS, until 4.0g of the adsorbent was consumed in TNT wastewater. With subsequent addition of adsorbent (5.0 g and beyond), the *COD* values plateaued at approximately 80 % decrease from initial *COD* values (Figure 9). This behavior revealed the amount of Fe_3_O_4_ NHS necessary for removing hazardous chemicals from the water stream to balance extraction efficiency and cost effectiveness. The schematic representation of the decrease of the *COD* values and change in color upon addition of Fe_3_O_4_ NHS is shown in (Figure 10).

### 3.7. Effect of pH and Initial Adsorbate Concentration

**Figure 11:**
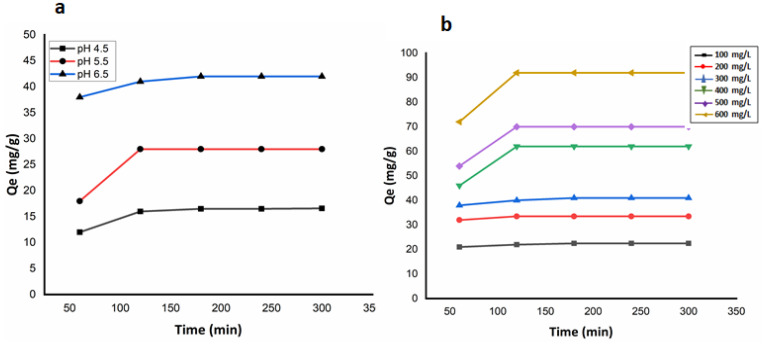
Effect of pH (**a**) and initial concentration of adsorbate (**b**) on the adsorption of TNT wastewater over the applied Fe_3_O_4_ NHS as an adsorbent.

The pH and concentration of the adsorbate (TNT wastewater) are the two important rate controlling parameters in calculating the overall adsorption efficiency of the applied adsorbent. To determine the optimum values of these parameters, a series of experiments were carried out varying pH and concentration values of the TNT wastewater, as shown in Figure 11a,b, respectively. It was observed that the applied Fe_3_O_4_ NHS works best at pH range 6–7 and no significant change was noted beyond pH 6.5. In a relatively more acidic environment, an excess of H+ ions compete with positive cations offered by the adsorbate, and thus decreases the adsorption of TNT wastewater. Figure 11a shows the extent of adsorption is minimum at pH 4 and increases with pH of the adsorbate until it reaches its maximum value of adsorption at pH 6.5, where the adsorbent performs well. Figure 11b shows the effect of the initial concentration of TNT wastewater against the constant weight of Fe_3_O_4_ NHS (1.0 g). At high concentrations of TNT wastewater, the rate of adsorption increases as the available active sites of Fe_3_O_4_ NHS are surrounded by the adsorbate cations due to the electrostatic interactions.

### 3.8. Adsorption Behavior

**Figure 12:**
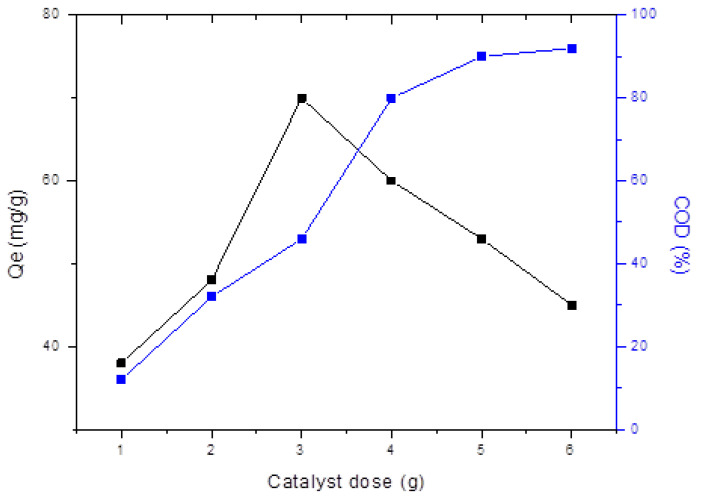
Effect of catalyst (Fe_3_O_4_ NHS) concentration on the adsorption capacity (*Qe*) and % decrease in *COD* values of TNT wastewater (TNT volume is 0.05 L at room temp. and pH value 6.5).

**Figure 13:**
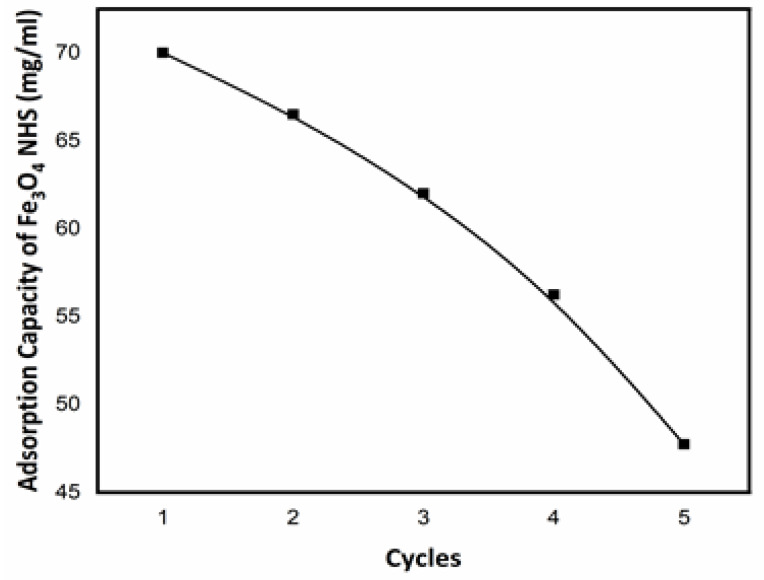
Decrease in adsorption efficiency (η) of Fe_3_O_4_ NHS (mg/g) over the number of cycles applied on TNT wastewater.

The overall adsorption capacity (*Qe*) of Fe_3_O_4_ NHS increased from 38 mg/g to 70 mg/g, when increasing the Fe_3_O_4_ NHS dose from 1.0 g to 3.0 g respectively, as shown in Figure 12. The initial adsorption of adsorbate is much faster, indicating that the adsorption rate increases with an increasing adsorbent dose until it reaches its optimum value of 70 mg/g, where equilibrium is established and a descending trend in adsorption is observed. This decrease in adsorption capacity is due to the occupied active sites over the surface of the Fe_3_O_4_ NHS. In contrast, the *COD* values of TNT wastewater decreased up to 92% in a gradual and steady fashion. Apart from the active sites’ chemistry, there exist other factors contributing to and facilitating this adsorption process. The inner and outer surface of the Fe_3_O_4_ NHS accommodates a large number of hydroxide (OH−) groups, creating a negative charge on its surface and increasing electrostatic forces, resulting in an increase in adsorption capacity of the adsorbent. It is also concluded that the negatively charged adsorbent (Fe_3_O_4_) possessed weakly attractive Van der Waals forces with TNT wastewater (positively charged), which is proposed as one of the dominant adsorption mechanisms. For calculating the adsorption efficiency (η) using (Equation (3)), Fe_3_O_4_ NHS was regained after changing the pH of the adsorbate solution and reapplying the adsorbent to the TNT wastewater. A decrease in (η) of Fe_3_O_4_ NHS after each cycle is shown in Figure 13. This decrease in adsorption efficiency (η) of Fe_3_O_4_ NHS was 15.12% after the 5th adsorption cycle.

## 4. Conclusions

This research work demonstrates a one-pot, hydrothermal, template-free method for the successful synthesis of α-Fe_2_O_3_ (hematite) and Fe_3_O_4_ (magnetite). Fe_3_O_4_ NHS were further used for the treatment of TNT wastewater. The variation in shape, size, morphology, porosity, and surface area was observed upon variation in temperature and time of the calcination in an autoclave. The increased surface area and high porosity associated with the Fe_3_O_4_ NHS yielded impressive results with regards to adsorption of TNT and other associated species with TNT wastewater. UV/visible spectroscopy results have confirmed the quick adsorption action of Fe_3_O_4_ NHS. The adsorbent Fe_3_O_4_ NHS was also tested as a function of contact time, dose, and temperature. In an industry where adsorption of different hazardous nitro-bodies like in TNT effluents is required, these magnetic NHS could have a significant impact. The synthesized Fe_3_O_4_ NHS effectively adsorbed nitro-bodies from the provided TNT effluent sample and decreased its COD values by 92 %, providing a safe environment for living and marine life in the aqueous environment. Better adsorption and recyclability in a shorter time-period gives NHS the benefits of increased efficiency and makes it a more economical option. The template-free hydrothermal synthesis, practical scale up options, and ease at which it can be employed, gives the advantage of applicability on an industrial scale. Considering all these advantages, this process is recommended for treating any industrial effluents generated from the production of cyclonite, cyclotetramethylene-tetranitramine (HMX), nitroglycerin (NG) production plants and other various environmental pollutants/species, which are hazardous for our environment and marine life.

## Figures and Tables

**Table 1:** BET isotherm tabular report for N_2_ adsorption over the surface of Fe_3_O_4_ NHS at evacuation rate of 1000.0 mmHg/min and saturation pressure of 760.0 (mmHg).

Relative Pressure (p/p°)	Quantity Adsorbed (cm^3^/g STP)	1/[Q(p°/p^−1^)]
0.0104	7.6735	0.0014
0.0503	10.5414	0.0050
0.2328	19.5293	0.0155
0.4552	31.1862	0.0269
0.6779	48.6297	0.0433
0.9004	145.6582	0.0621

**Table 2:** BET surface area report for N_2_ adsorption over the surface of Fe_3_O_4_ NHS at evacuation rate of 1000.0 mmHg/min and saturation pressure of 760.0 (mmHg).

Relative Pressure (p/p°)	Absolute Pressure (mmHg)	Quantity Adsorbed (cm^3^/g STP)	Elapsed Time (h:min)	Saturation Pressure (mmHg)
0.0104	7.8496	7.6735	00:42	760.00
0.0503	38.2094	10.5414	00:45	
0.2328	176.8626	19.5293	00:46	
0.4552	345.8628	31.1862	00:48	
0.6779	515.1983	48.6297	00:50	
0.9004	684.2429	145.6582	00:55	

## Data Availability

All the data will be available to the readers.
